# Provocation of attacks to discover migraine signaling mechanisms and new drug targets: early history and future perspectives - a narrative review

**DOI:** 10.1186/s10194-024-01796-1

**Published:** 2024-06-20

**Authors:** Jes Olesen

**Affiliations:** Danish Headache Center, Department of neurology, Rigshospitalet- Glostrup, University of Copenhagen, Valdemar Hansens Vej 5, Glostrup, 2600 Denmark

**Keywords:** Migraine, Headache, NO, CGRP, PACAP, Histamine, Headache models

## Abstract

**Introduction:**

The development of several experimental migraine provocation models has significantly contributed to an understanding of the signaling mechanisms of migraine. The early history of this development and a view to the future are presented as viewed by the inventor of the models.

**Methods:**

Extensive knowledge of the literature was supplemented by scrutiny of reference lists.

**Results:**

Early studies used methodologies that were not blinded. They suggested that histamine and nitroglycerin (Glyceryl trinitrate, GTN) could induce headache and perhaps migraine. The development of a double blind, placebo-controlled model, and the use of explicit diagnostic criteria for induced migraine was a major step forward. GTN, donor of nitric oxide (NO), induced headache in people with- and without migraine as well as delayed migraine attacks in those with migraine. Calcitonin gene-related peptide (CGRP) did the same, supporting the development of CGRP antagonists now widely used in patients. Likewise, pituitary adenylate cyclase activating peptide (PACAP) provoked headache and migraine. Recently a PACAP antibody has shown anti migraine activity in a phase 2 trial. Increase of second messengers activated by NO, CGRP and PACAP effectively induced migraine. The experimental models have also been used in other types of headaches and have been combined with imaging and biochemical studies. They have also been used for drug testing and in genetic studies.

**Conclusion:**

Conclusion. Human migraine provocation models have informed about signaling mechanisms of migraine leading to new drugs and drug targets. Future use of these models in imaging-, biochemistry- and genetic studies as well as in the further study of animal models is promising.

## Introduction

Fifty years ago, migraine mechanisms were largely unknown, and there was no clue how to understand them. The situation was at that time often compared to psychiatry. Increased urinary excretion of 5-hydroxy-indole-acetic acid (5-HIA) during migraine attack was a glimpse of hope, but the role of 5-HT in migraine has proven difficult to understand even after introduction of the 5-HT1 receptor agonists, the triptans [1]. There was, and still is, no pathoanatomical substrate and no chemical biomarkers in migraine which, therefore, must be a disease of abnormal function, most likely abnormal signaling. At that time it was, however, difficult to study the signaling mechanisms of migraine.

The old literature (before 1980) contained many reports of headache provoked by various chemicals such as nitroglycerin and histamine, but was it a psychological effect? In the absence of studies with a controlled double-blind design this was uncertain. Migraine patients were probably more sensitive to these chemicals than others, but in the absence of diagnostic criteria it was uncertain, if it was migraine that was provoked or just a headache unrelated to migraine. In the nineteen eighties it was tempting to deliberately try to provoke headache/migraine in patients, but would it be ethically acceptable? It had been done before but that was before ethical committees. The attitude to human experimentation had become different in the nineteen eighties. In virtually all other diseases, it would be unethical and unacceptable. Migraine was, however, different. It is characterized by self-limiting attacks and therefore provocation was not likely to elicit chronic headache. Furthermore, attacks cause no physical damage even if they are very painful and debilitating. Finally, attacks can be effectively treated as soon as the experimental subject so wishes. It should be up to each patient if they are willing to sustain a provoked attack. These considerations led to ethical acceptance of provocation and the development of a human provocation model of migraine [2]. It has now been used for 30 years in an ever-increasing number of scientific studies without any serious adverse events. Studies with this human provocation model have greatly improved our understanding of the signaling mechanisms of migraine.

The provocation model was developed over several years, but thereafter its main features have remained essentially unchanged. It has been combined with ultrasound imaging of relevant extracerebral arteries, transcranial doppler of brain arteries and blood pressure and heart rate measurements. The techniques of measurement have gradually been refined, particularly by adding various MR and PET techniques. In addition to evaluating disease mechanisms, the model has also been used to determine therapeutic responses, nociceptive mechanisms, differences between migraine subtypes and influence of genetics. The human provocation model has also inspired the development of rodent models of migraine. The human migraine provocation model will most likely be used increasingly also outside of its main home, the Danish Headache Center. It is on this background that the founder of the method here describes the history of this invention and the major early results. Some of the expected future possibilities are also briefly discussed.

## Methods

Literature lists of personal and other known publications were searched for suitable references. The search was limited to English language publications. Reviews were selectively included.

### Studies before the modern human provocation models

#### Nitroglycerin

Antonio Sobrero who synthetized nitroglycerin, or glyceryl trinitrate (GTN) as it was later called, described a violent headache after its intake [3]. Subsequently headache after GTN was described by several others. In the dynamite industry, where GTN was an ingredient, workers frequently complained of headache [3]. Dermal application was studied using a GTN containing ointment in the belief that it worked locally in the temple. However, we now know that it is readily absorbed through the skin and probably worked systemically. GTN ointment was also used to compare migraine sufferers to controls. In a study using doses of 1,2,4 and 6 mg, the 2 mg dose distinguished 100% between subjects with migraine and normal subjects [4]. The study was not blind or randomized and, in a double-blind trial using only the 6 mg dose [5], the overlap between patients and controls was too big for the test to be useful in the diagnosis of individual cases. Peters [6] described a delayed migraine-like headache after GTN in 1953 and Sicuteri [7] confirmed its existence in 1987. None of these studies were double-blind. Neither did they have available the explicit diagnostic criteria for migraine of the International Classification of Headache Disorders (ICHD-1) [8] for comparison. The mode of administration of GTN involved a large variation in absorption. Together these and other limitations left many questions open before an experimental headache provocation with GTN was optimal.

#### Histamine

Pickering and Hess, following several earlier observations by others, published a monumental study of headache induced by single intravenous injection of histamine [9]. The subjects were not characterized with regard to headache diagnosis, but it was noted that those who had a history of previous headache got a stronger headache. Dose finding suggested that 0,1 mg of histamine i.v. was the strongest still well-tolerated dose. Headache and blood pressure were measured at frequent intervals. Blood pressure decreased first and, after its recovery 60 s after the injection, headache began. Before headache a sensation of throbbing in the head was often noted. Shaking the head made the headache worse while compression of peri cranial tissues with an inflatable cuff did not relieve it. Histamine caused increased intracranial pressure and increased intracranial pulsations measured by lumbar puncture. Headache was thought to originate from the meninges. In summary, many older studies demonstrated beyond doubt that this naturally occurring monoamine very effectively induced headache and that there might be an increased response in persons with a previous headache disorder.

#### Meta-chlorphenylpiperazine (m-CPP)

M-CPP stimulates the release of cortisol and prolactin. In a study of its effect on eating disorders, it was observed that many patients developed a migraine-like headache [10]. Subsequently patients were questioned about previous headaches and a correlation to previous migraine was described. However, a subsequent prospective study comparing subjects with migraine and normal controls did not find a significant difference although both groups developed headache [11]. M-CPP also binds to the 5-HT2C receptor in the endothelium and activates NO synthase and NO production [12]. This may be the reason for the induced headache. Because the compound has so many different actions in the body its use in further provocation experiments is not likely to clarify signaling mechanisms.

#### Reserpine

Reserpine causes depletion of platelet serotonin and other compounds starting almost immediately and reaching its maximum value after 5–7 h. The development of headache seems to roughly parallel the depletion of serotonin but with considerable variation. Patients have reported that the headache is like their usual migraine attacks [13, 14]. All published studies were relatively small, not blinded and without sufficient clinical information to diagnose experimental migraine. Nevertheless, these studies supported the development of sumatriptan. Given the marked biochemical changes in blood after reserpine it seems tempting to combine reserpine induced migraine with modern transcriptomics and metabolomics.

## The modern human provocation models

### Background to the modern human models

It is extremely difficult to study migraine mechanisms because patients are normal interictally and because they prefer to stay at home during attacks. Even when they are willing to come to the hospital, they arrive at variable times of the day and a variable number of hours after onset of the attack. If investigations include advanced equipment, it must be freely available. For these reasons studies of spontaneous acute migraine attacks usually include only single cases or a very small number of participants. Carotid angiography was the primary mode of investigation of cerebral disorders in the 60s and 70s before CT scanners. Surprisingly, angiography induced migraine aura and that made it possible to record changes in regional cerebral blood flow before and during the onset of a migraine with aura attack. The results strongly indicated that cortical spreading depression is the mechanism underlying the migraine aura [15]. This kind of migraine provocation was unexpected and involuntary, but it demonstrated that provocation of migraine attacks was valuable to study the disease. Pharmacologically provoked attacks could be planned in time, and they would allow study of the initial phases of the migraine attack as opposed to spontaneous attacks. It would be possible to apply even very advanced study techniques such as MR-scanning and PET-scanning. First and foremost, provocation would prove a relation between the offending molecule and migraine. Thus, it would be possible to test different chemical entities, preferably signaling molecules present in the human body or compounds with a known specific mode of action. Histamine was selected as the first compound to study in view of the previous publications on histamine provocation, and because it was a naturally occurring signaling molecule present in many tissues of relevance to migraine. Persons with migraine, others with tension-type headache and headache free control persons were diagnosed as precisely as possible before the availability of the International Classification of Headache Disorders (ICHD-1). Histamine was given as a continuous intravenous infusion [16]. Migraine patients but not controls developed a throbbing headache. Mepyramine, a histamine H1 receptor blocker, completely abolished the headache during continued infusion of histamine. There was a small but significant effect of cimetidine, a H2 blocker. This study had several new features such as precise headache diagnoses, continuous IV infusion of the provoking agent to eliminate variability of absorption and testing of antagonists. It was not double-blind, however, and the only way to prove that migraine patients developed migraine attacks was by asking for similarity with previous attacks. Explicit diagnostic criteria that allowed an objective scoring of the induced migraine became available only with the International Classification of headache Disorders (ICHD-1) in 1988.

### Development of the standard provocation model

**Fig. 1:**
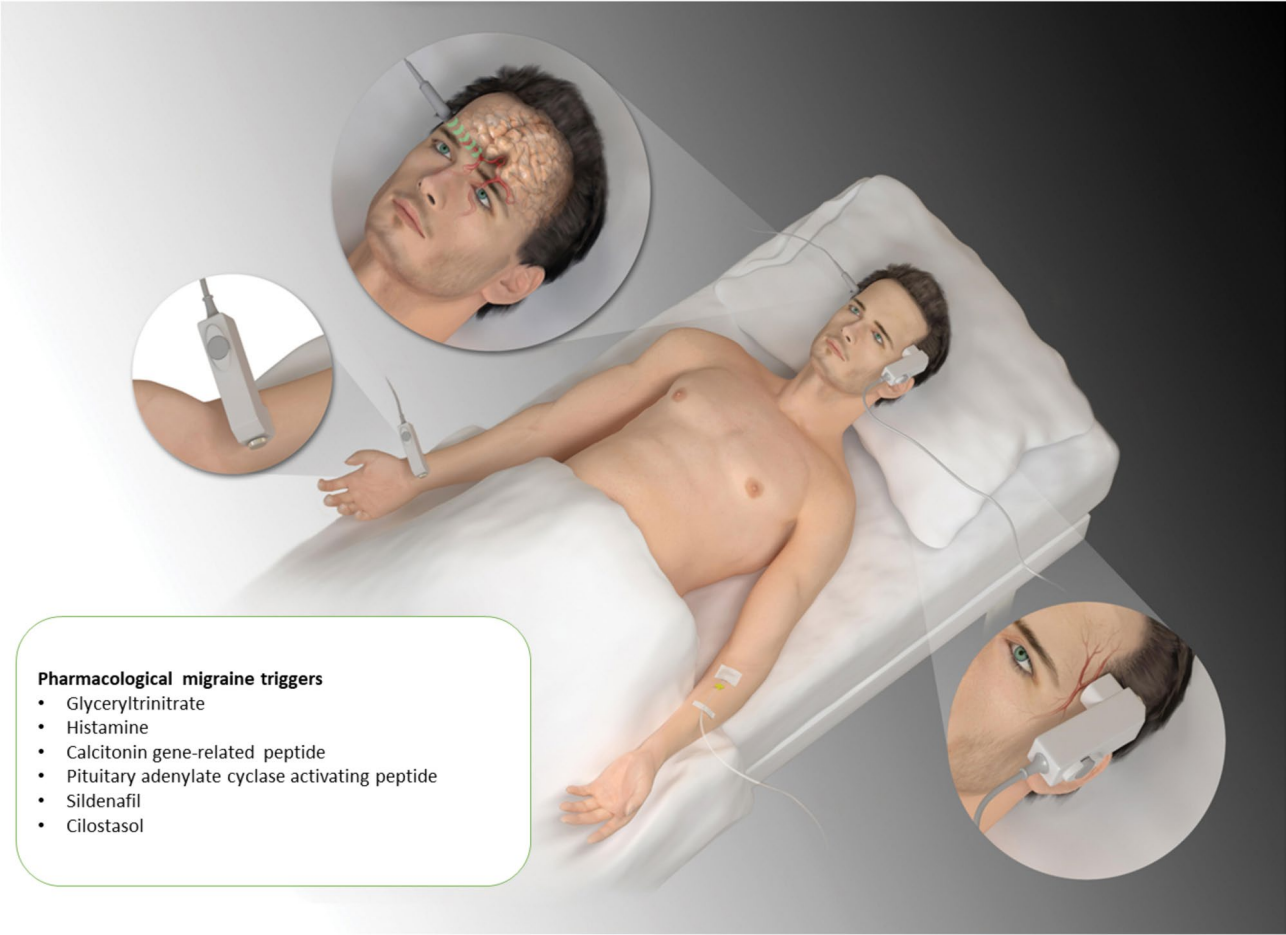
The human provocation model involves IV infusion of a provoking substance (see insert) usually for 20 min. Headache, blood pressure, heart rate and the diameter of superficial temporal- and radial arteries and velocity of blood in the middle cerebral artery are monitored at short intervals. After discharge a headache diary is kept every hour until 12 h after the provocation. Many substances cause migraine in this model.Modified from previous publications

**Fig. 2:**
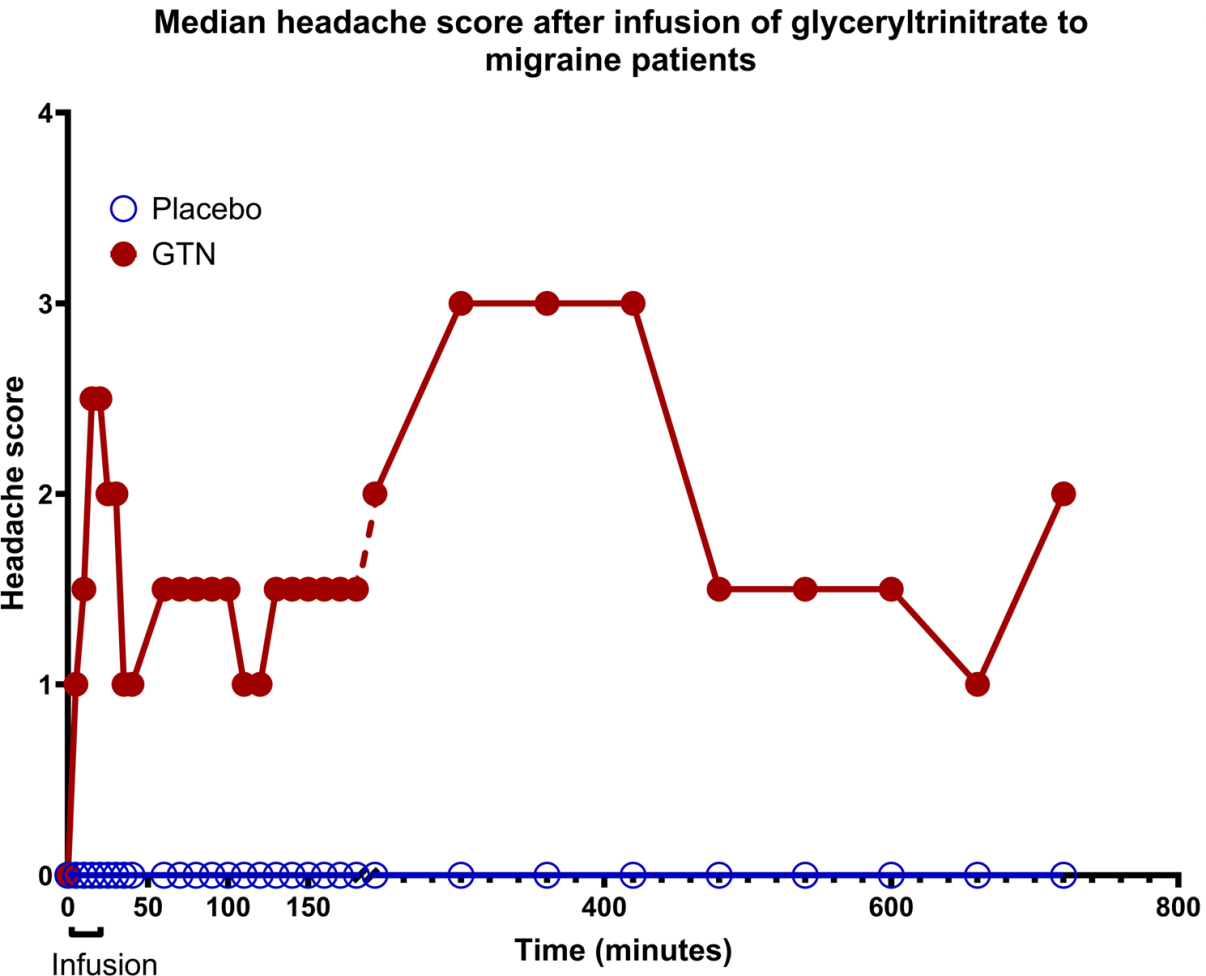
The provocation model was fully developed using glyceryl trinitrate (GTN) infusion 0,5 µg/kg/min for 20 min. Headache is scored 0–10. There is an immediate headache during infusion, regression towards normal and then a delayed headache during which diagnostic criteria for an attack of migraine without aura are fulfilled. The lag phase between immediate and delayed headache has also been observed with other substances. Modified from [2]

Based on the above experience we continued with the development of what became the standard provocation model (Figs. 1 and 2)^2^. We chose GTN for the model development because of its known ability to cause headache and probably also migraine. First a dose finding study was done infusing GTN twice intravenously in 4 different doses VS placebo to persons without migraine in a double-blind fashion [17]. Headache was maximal at a dose of 0,5 µg/kg/min and higher doses caused no more headache (ceiling effect). The reproducibility of the induced headache was good. Headache reached a maximum in 2–5 min and came back to baseline 10–20 min after end of the infusion in these healthy persons. Clearly, 10 min was too short an infusion time for a useful steady state headache. The next study compared migraine to tension-type headache (TTH) and controls. Since the effect in persons with migraine was unknown, we used infusion with stepwise increasing doses of GTN [18]. Patients with migraine without aura (MO) got more headache than headache free controls. Headache fully remitted after infusion in controls but not quite in MO patients. After discharge most MO patients but not controls developed an attack fulfilling ICHD-1 diagnostic criteria for MO. Based on these experiences the next development of our method used GTN 0,5 µg/kg/min or placebo in MO patients. It was infused intravenously to avoid absorption variability and we used a longer infusion time of 20 min. The design was double-blind comparing GTN to placebo. After GTN 8 of 10 patients got a biphasic headache (Fig. 2). No biphasic headache developed after placebo. The immediate headache during and shortly after the infusion had some migraine characteristics, but it did not fulfill ICHD-1 diagnostic criteria for a migraine attack without aura available for a few years. The delayed headache which had its maximum around 5.5 h after infusion occurred only in persons with migraine. It fulfilled ICHD-1 diagnostic criteria for an attack of MO. A biphasic headache with an immediate component not fulfilling migraine criteria and a delayed headache that does fulfill migraine criteria has also been found after provocation with other substances such as histamine, CGRP and PACAP. It is seen in most migraine subjects but almost never in persons without migraine.

When studying a novel molecule, we first do a pilot dose-finding study in normal persons. Then a double-blind study in normal persons and finally, a study in persons with migraine. Normally there is sufficient published information to roughly estimate the necessary dose. Therefore, only two to four doses need evaluation in the pilot experiment [19]. The next study also uses normal subject. In a cross-over experiment comparing the presumed active dose to placebo double-blindly, the experience has been that substances causing a headache in normal subjects very often cause migraine in persons with migraine. The headache inducing substance is then tested in migraine subjects, preferably in a cross-over design comparing the substance to placebo. Early on, reviewers insisted on studies comparing migraine patients to healthy controls, but decades of experience have demonstrated that no more than 10% of migraine patients develop a migraine-like headache after placebo [20]. This nocebo effect is much smaller than we and others previously expected. The stress of the experimental situation does not induce migraine attacks and comparison to normal subjects is usually not necessary. Once the migraine inducing ability of a novel compound has been documented, focus is on its possible biological mechanisms in the human body. They can be investigated during induced attacks by blood chemistry including transcriptomics, metabolomics, proteomics, various MR modalities and PET. For these expensive studies, attack may be compared to baseline of the same patient although a control group is always better. Provocation may also be useful in pharmacological studies as discussed below.

### Further studies of GTN and other NO donors

During development of the provocation model, it was shown by others that GTN is a pro-drug for NO, and that NO is the so called endothelial relaxing factor. The conversion of GTN to form NO was known to be facilitated by N-acetylcysteine. In a double-blind provocation study in normal subjects N-acetylcysteine augmented GTN induced headache and dilatation of the superficial temporal artery but not of the radial artery [21]. Isosorbide mononitrate (ISMN), a long acting NO donor, caused a prolonged headache in normal subjects [22]. Similarly, infusion of GTN for 7 h resulted in headache and arterial dilatation for more than 7 h [23]. Together these studies strongly suggested that GTN caused headache due to its delivery of NO to the organism. This was later confirmed, as sodium nitroprusside also caused headache [24].

NO has a very short half-life so secondary mechanisms induced by NO must be implicated to explain the delayed migraine. NO starts a cascade of reactions activating soluble guanylyl cyclase, protein kinase G (PKG) and ATP sensitive potassium channels (K_ATP_) [25]. It remains uncertain if this can explain the time lag between immediate and delayed headache. The super sensitivity to GTN in MO patients could be an effect of frequent attacks or of genetic disposition. There was no difference in the GTN response between patients with rare attacks and patients with frequent attacks [26]. Therefore, super sensitivity to NO was probably genetically determined. The super sensitivity had been demonstrated in MO. In a group of patients with pure migraine with typical aura (MA), who had never had an attack without aura, GTN caused no aura, but the patients developed an attack fulfilling criteria for MO for the first time in their life [27]. It is still not understood why patients with MA and no MO attacks respond in this way. The numbers in this study were small but subsequent studies demonstrated almost identical figures in larger numbers of patients [28, 29]. Familial hemiplegic migraine (FHM) patients did not develop an attack after GTN neither of aura nor of M0 [30]. This remarkable biological difference between FMH, MO and MA suggests that rodent models of FMH may not be completely relevant for MO or MA.

Is the migraine inducing effect of GTN in the brain or outside? GTN diffuses freely across membranes including the blood brain barrier and gets into the brain, but it is unclear to which extent brain tissue other than blood vessels can convert GTN to NO. A double-blind trial showed that GTN only altered peripheral pain thresholds in an area overlying the temporal muscle and not in two other locations suggesting that it causes nociception locally [31]. This was further strengthened by the fact that GTN causes no central side effects such as sedation or dizziness. Another study suggesting a peripheral mechanism of GTN examined the well-known development of tolerance. In a randomized double-blind crossover design, 11 healthy subjects received 30 mg ISMN 3 times daily or placebo for 7 days. Washout between periods was 14 days or more [32]. With this heavy continuous NO challenge, 10 healthy subjects fulfilled the pain sub criteria for MO and 5 subjects fulfilled all diagnostic criteria including those for accompanying symptoms. The experiment suggested that normal individuals can develop a migraine attack given a sufficiently strong provocation. There was a close temporal relationship between the disappearance of headache and the disappearance of dilatation of the superficial temporal artery. Both took approximately 72 h while tolerance in the middle cerebral artery developed already after 24 h long before tolerance to headache. But in favor of a cerebral site is the finding of premonitory symptoms after GTN provocation [28]. The super sensitivity to NO in migraine sufferers was not only shown for headache but also for blood velocity in the middle cerebral artery [33, 34]. The effect of GTN is not dependent on histamine because blockade of the H1 receptor with mepyramine had no effect on GTN induced headache [35].

One thing is that NO can induce a migraine attack but are NO related mechanisms also active throughout the entire attack? This question was addressed in a double-blind study of L-NMMA which is a non-selective blocker of all three enzymes that produce NO in the organism [36]. It was significantly more effective than placebo suggesting that endogenous NO production is likely to be continuously increased during spontaneous migraine attacks. Therefore, antagonists against NO or its downstream cascade may be effective treatment of migraine.

If an experimental migraine model could be used to test potential novel drugs for migraine, it would facilitate their development. It would be most useful for prophylactic drugs because they are more difficult and expensive to develop than drugs for the acute attack. In the standard migraine provocation paradigm with GTN given intravenously for 20 min, it was examined whether prophylactic treatment with propranolol or valproate could diminish the headache/migraine. Propranolol had no effect [37], but valproate significantly decreased the induced headache compared to placebo [38].

Treatment of acute attacks has also been studied in the GTN model. The experimental compound tonabersat was compared to placebo in a double-blind crossover trial in MO patients. Both arms received GTN provocation followed by tonabersat or placebo [39]. Tonabersat had no effect. Likewise, the CGRP receptor antagonist olcegepant given as pretreatment before GTN provocation in a double-blind placebo-controlled crossover study of MO patients had no effect [40]. Sumatriptan given as injection slightly, but significantly reduced GTN induced headache in normal volunteers [41, 42]. These results were confirmed by Fullerton et al [43]. Prednisolone did not decrease immediate GTN induced headache but decreased delayed GTN induced migraine in MO patients [44]. Migraine subjects are difficult to recruit for this kind of experimental study.Therefore it was investigated whether a model in normal volunteers could be used in future drug trials. A double-blind trial, where GTN was given in a smaller dose of 0,25 µg/kg/min for 2 h and 20 min, showed no effect of oral aspirin or zolmitriptan compared to placebo [45]. In summary, the GTN model may in some situations respond to migraine treatment, but the model is most often not useful in migraine subjects or normal volunteers. Sensitivity and specificity cannot be calculated from these heterogenous studies, but further studies of larger patient materials seems warranted.

The GTN model was used to evaluate why a few persons in the general population never ever have had a headache [46]. The usual provocation model compared such individuals to matched randomly selected persons from the normal population. There was no difference, and persons who had never had a headache before experienced their first headache. It was concluded that freedom from headache is not explained by alterations in NO mechanisms but it remains to be seen, if it can be explained by other provoking molecules.

The GTN model has also been used in persons with tension-type headache [47] who showed increased sensitivity compared to normal individuals as well as a delayed headache fulfilling diagnostic criteria for tension-type headache. GTN also induced attacks of cluster headache during a cluster period. A detailed account of the effect of GTN in headaches other than migraine is outside of the scope of this paper, but NO seems to be involved in TTH and cluster headache.

### Histamine

The early studies of histamine cited above had left some uncertainty because they were not double-blind, and patients were not classified according to ICHD-1. After development of the migraine/headache provocation model to include double blind intravenous infusion for 20 min, and the diagnostic criteria for migraine of the ICHD-1, it was decided to study histamine again. 20 MO patients received pretreatment with placebo or the histamine H1-receptor antagonist mepyramine in a randomized double-blind fashion followed in both groups by IV histamine (0,5 µg/kg/min for 20 min) [48]. In patients given placebo histamine caused immediate headache during the infusion followed by a delayed migraine attack fulfilling IHS criteria for a migraine without aura attack. The temporal profile of induced headache was the same as in previous studies of GTN. Mepyramine pretreatment abolished both immediate headache and delayed migraine. It was concluded that histamine likely induced migraine via activation of endothelial H1-receptors leading to formation of NO. This could not be confirmed, however, in other studies where patients were pretreated with the non-selective nitric oxide synthase inhibitor L-NMMA or placebo followed in both groups by histamine infusion [42, 49]. There was no effect of L-NMMA on the histamine induced headache. In both groups histamine decreased middle cerebral artery (MCA) velocity and dilated the superficial temporal artery 4–5 times more than the radial artery. These vascular effects were also not inhibited by L-NMMA. In conclusion histamine induced headache and migraine are either independent of NO formation or the L-NMMA infusion had not been sufficient to block the ligand induced formation of NO.

Several studies had suggested that headache patients developed more headache after histamine injection than persons without a history of headache^9^. Also, open use of GTN provocation as a diagnostic tool was positive^4^. Histamine inhalation was used widely in the diagnosis of asthma and its ability to add to the migraine diagnosis was now studied. 15 migraineurs and 15 control subjects inhaled increasing doses of histamine [50]. Headache increased dose dependently and 6 migraine patients, but no controls developed a migraine attack. Thus, the specificity of the test was excellent, but the sensitivity was only 0,4. Although histamine is highly effective in inducing migraine attacks, antihistamines have generally not been effective in the treatment of migraine. The possibility of involvement of the H3-receptor has been discussed [51] but seems unlikely because of the almost total efficacy of histamine H1-receptor blockade with mepyramine. Thus, histamine is so far the only example of a migraine provoking agent where an antagonist is known not to be effective in the treatment of spontaneous migraine. In future this may likely be true also for other migraine provoking agents where antagonists have not yet been tested for efficacy.

### Calcitonin gene-related peptide (CGRP)

**Fig. 3:**
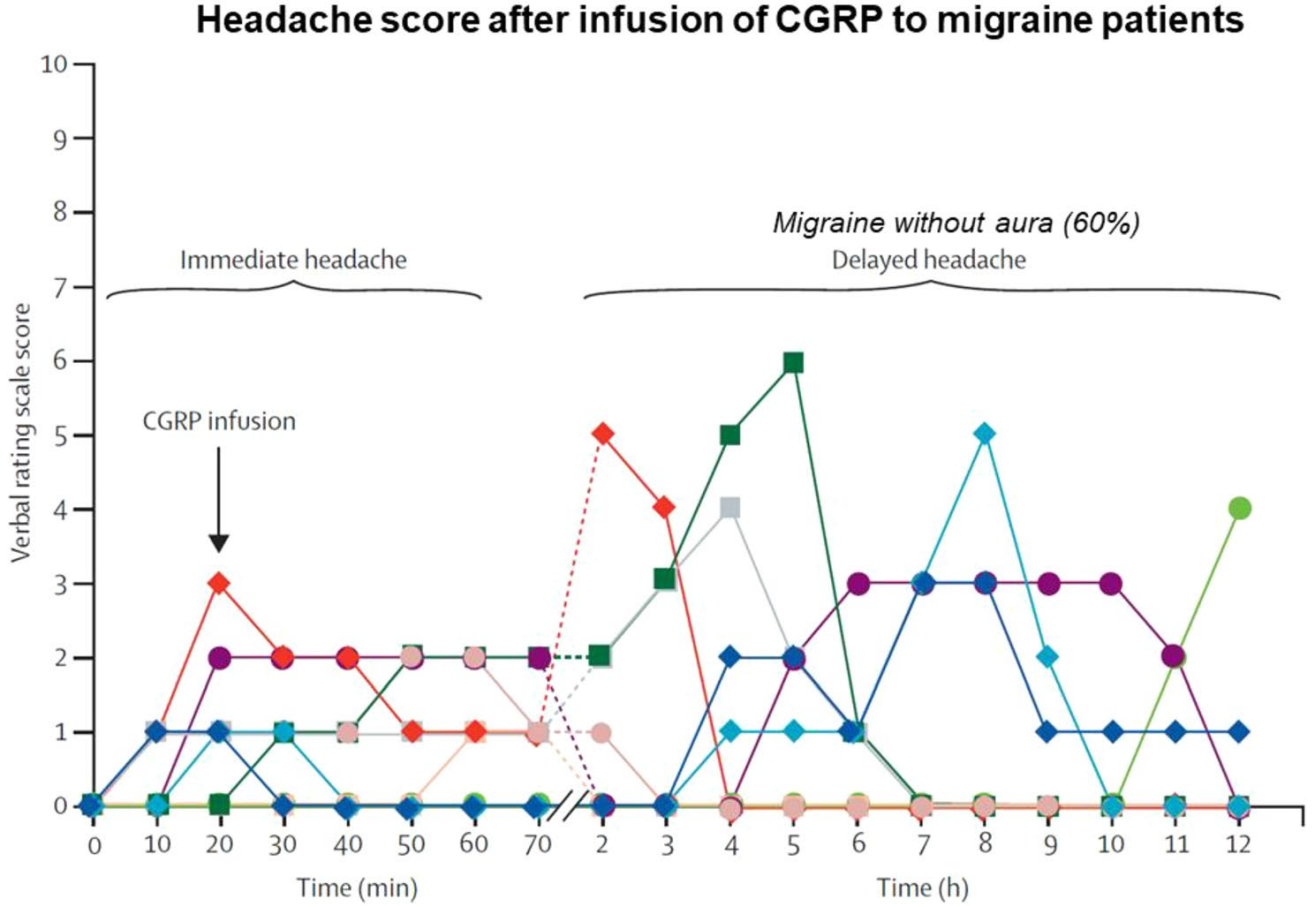
The provocation model applied to calcitonin gene-related peptide (CGRP). Headache is scored 0–10. One color per patient. An immediate headache was followed hours later by a delayed headache fulfilling migraine diagnostic criteria in 60% of the cases. Modified from [54]

Out of the many signaling molecules disclosed by human provocation to have a role in migraine, CGRP is by far the most important. The localization of CGRP to migraine relevant tissues, its effect as a strong vasodilator particularly in cranial blood vessel, the liberation of CGRP from activated trigeminal nerve terminals and the provocation of migraine attacks by CGRP infusion in patients led to the development of novel CGRP antagonizing drugs for acute migraine attacks and for migraine prophylaxis. This has been extensively reviewed previously [52–54]. Here we shall concentrate on human provocation studies with CGRP. In a double-blind study Lassen et al. demonstrated that 20 min infusion of CGRP (2 microg/min/kg) to persons with MO induced significantly more headache than in a normal control group [55]. Several migraine patients developed an attack fulfilling ICHD-1 criteria for migraine without aura (Fig. 3).

The data were presented to Boehringer-Ingelheim in 1995 and strongly supported the ongoing development of the first CGRP receptor antagonist olcegepant. Due to illness they were, however, not fully published until 2002. CGRP crosses the blood brain barrier poorly and it causes no change in regional cerebral blood flow or the velocity of blood in the middle cerebral artery [56]. In agreement, studies of isolated cerebral blood vessels showed that CGRP is a strong vasodilator given extra-luminally i.e. circumventing the blood brain barrier, but not given luminally when it had to cross the barrier at the endothelium [57]. Thus, CGRP mechanisms of migraine are likely outside of the brain. This paved the road for the development of CGRP antagonists that also hardly cross the blood- brain barrier [53]. CGRP is not only important in the brain or the extracranial tissues but throughout the body, most notably the heart. It is one of the strongest vasodilating agents known, and therefore it was feared that blocking CGRP might lead to vasospasm and ischemia throughout the body. This turned out not to be the case because a double-blind study of olcegepant, the first CGRP receptor blocker available for human use, had absolutely no effect on blood pressure, heart rate, velocity of blood in the middle cerebral artery or on the diameter of the superficial temporal artery or radial artery [58]. The dose of olcegepant given was four times higher than the effective dose treating a migraine attack. It was biologically highly efficacious because it totally blocked the effect of infused CGRP in another study [59]. Thus, antagonism of CGRP would be a safe approach to the treatment of migraine. CGRP could induce MO but what about other types of migraine? In a double-blind study Hansen et al. showed that CGRP infusion to patients who had migraine with typical aura (MA) resulted in attacks fulfilling criteria for an induced attack of MO [60]. Only in a few cases was CGRP followed by MA. When CGRP infusion was tested in patients with familial hemiplegic migraine, no attack was induced of FMH, MA og MO [61, 62].

Given that CGRP induces migraine attacks, the CGRP provocation model might be a useful tool to test novel drugs. In normal volunteers CGRP infusion resulted in mild headaches that were not reduced by pretreatment with sumatriptan [63]. When CGRP was infused in normal individuals for 2 h instead of the usual 20 min it did not result in more headache and the very mild induced headache did not respond to sumatriptan [64]. There were more side effects, however, especially gastrointestinal hyperactivity which made further studies of long-lasting CGRP infusion unattractive. In another study CGRP infusion caused no premonitory symptoms, again underlining that CGRP does not cross the blood brain barrier [65]. Similar results were seen using functional MR [66]. The bold signal was unchanged after CGRP but CGRP dilated the middle meningeal artery [67]. Many more studies have subsequently been done with CGRP to understand its importance in migraine, but they will not be reviewed here.

### Pituitary adenylate cyclase activating peptide (PACAP)

PACAP is a signaling peptide in the same family as CGRP. It was known to also be vasodilating and to act on 3 different receptors. It is not liberated during a migraine attack in contrast to CGRP. The whole story of PACAP and migraine has recently been summarized [68].

PACAP exists in two isoforms PACAP-38 and PACAP-27. They generally have the same effects. PACAP-38 is the most abundant and dominates in the head. PACAP-27 is primarily located in the enteric system [68]. PACAP-38 dilated the superficial temporal artery more than the radial artery in normal subjects but did not change the velocity of blood in the middle cerebral artery indicating no effect on the brain [69]. PACAP induced more headache than placebo in normal volunteers. This led to a study in persons with MO [70]. Migraine was induced significantly more often than after placebo. The dilatory effect of PACAP-38 was subsequently shown to be long-lasting, including dilatation of the middle meningeal artery [71]. This contrasted with the effect of the closely related peptide vasoactive intestinal polypeptide (VIP), which had only a short-lasting vasodilatory effect [72]. Although VIP caused headache, it did not cause a migraine-like headache significantly more often than placebo. The sensitivity to PACAP-38 was not genetically determined comparing migraine persons with and without familial aggregation of migraine [73]. In the same study an analysis of the possible biochemical mechanisms of PACAP induced migraine did not result in abnormal findings [74]. In agreement with existing data on the minute passage of PACAP into the brain [75], the lack of induction of premonitory symptoms and unchanged middle cerebral artery velocity suggested that PACAP acted outside of the blood-brain barrier. The skin reaction to local injection of PACAP-38 and VIP showed increased flow, wheal and flare of both compounds and thus, did not explain why PACAP-38 and not VIP caused migraine [76]. In a study of long-lasting infusion [77] migraine was provoked by VIP. The duration of vasodilation thus seemed responsible for migraine provocation. In a rodent model the longer lasting effect of PACAP was explained by its ability to degranulate mast cells [78]. PACAP-27 like PACAP-38 induced migraine-like headache [79].

### Downstream effects of NO, CGRP and PACAP

**Fig. 4:**
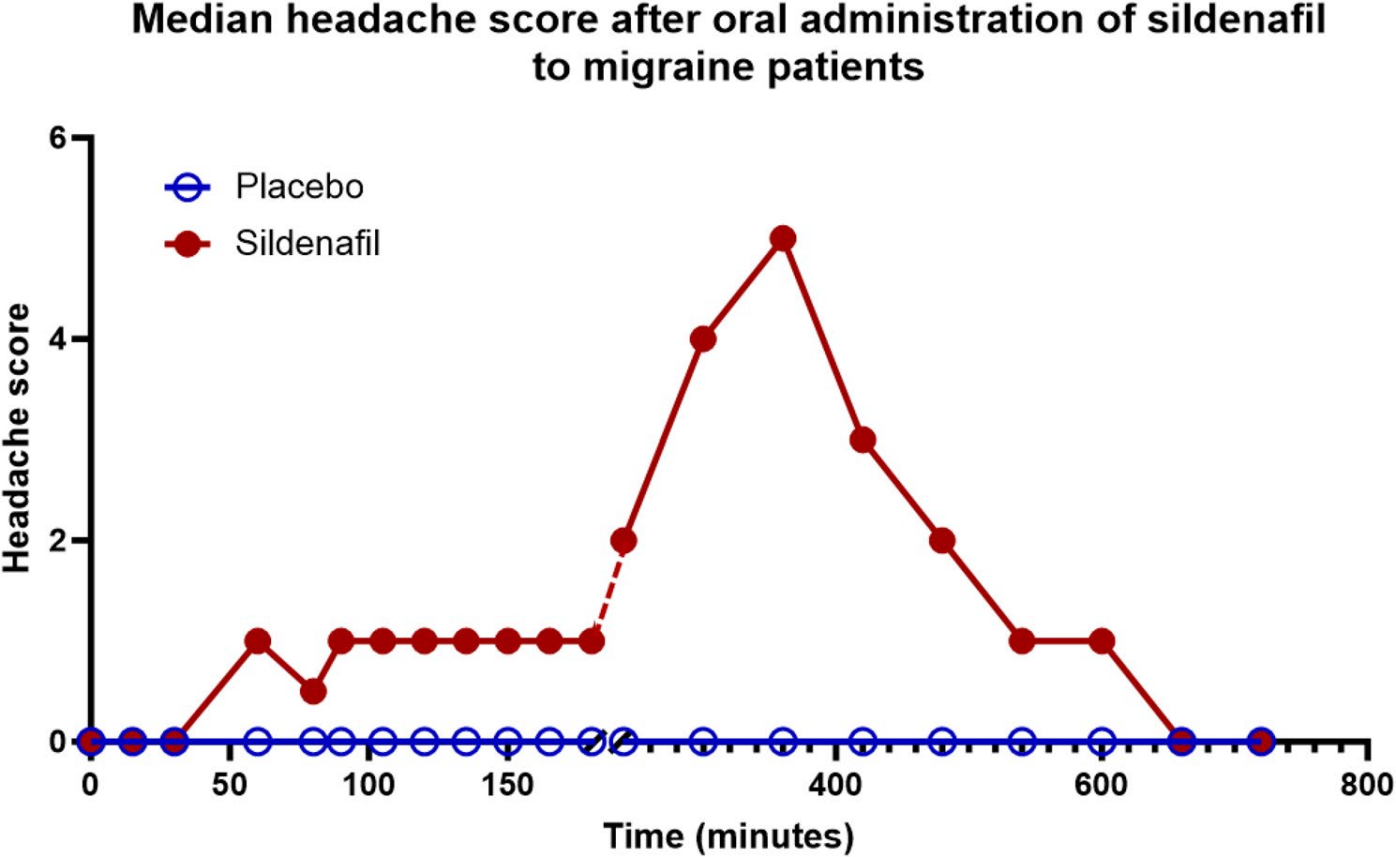
The headache provocation model using sildenafil, an inhibitor of phosphodiesterase 5 orally. Headache is scored 0–10. The curve is monophasic without distinction between immediate and delayed headache. However, after hours the headache fulfilled diagnostic criteria for a migraine without aura attack. Modified from [82]

NO has many different actions. The most important is its stimulation of soluble guanylyl cyclase to form increased amounts of cGMP but NO also binds to other iron containing enzymes and it forms peroxynitrite, a free radical (Fig. 4). In animal experiments the latter pathway plays an important role [80]. In humans, the question was studied using sildenafil which is an inhibitor of Phosphodiesterase-5 (PDE-5) whichbreaks down cGMP. When PDE-5 is inhibited, cGMP accumulates. Sildenafil caused headache in normal subjects significantly more often than placebo [81] and migraine attacks in 83% of persons with migraine [82]. This is the same frequency as seen after GTN. The conclusion was that the NO-cGMP pathway is by far the most important in human migraine.

Activation of CGRP and PACAP receptors as well as receptors for prostaglandins and several other migraine provoking signaling molecules leads to activation of soluble adenylate cyclase and the formation of cyclic AMP (cAMP). It cannot be ruled out, however, that activation of these receptors have other effects in the cell. Therefore, it was tempting to directly increase cAMP and compare the response to that of CGRP and PACAP. Cilostazol inhibits phosphodiesterase 3 which is the main enzyme degrading cAMP. When degradation is inhibited or abolished, cAMP accumulates. Cilostazol was first studied in normal volunteers, and it caused significantly more headache than placebo [83]. Furthermore, it dilated not only extracerebral arteries but also the middle cerebral arteries. It had no effect on cerebral blood flow. In persons with MO cilostazol caused a migraine-like attack in 86% of cases versus placebo in only 14% [84]. Thus, cilostazol, if anything, was more effective causing migraine than CGRP and PACAP. Therefore, it was highly likely that the only effect of CGRP and PACAP leading to a migraine attack was the stimulation of cAMP production. Thus, NO, CGRP and PACAP all seem to induce migraine attacks via augmentation of their respective second messengers cGMP and cAMP.

## Future perspectives

This review focuses on the early history of migraine provocation with histamine, GTN, CGRP and PACAP. Many subsequent studies led by Messoud Ashina have demonstrated that prostaglandins, VIP, amylin and adrenomedullin also can induce migraine, but will not be reviewed here. For all these substances and to some extent also for NO, CGRP and PACAP, downstream mechanisms need to be further clarified in future human studies and, when that is not possible, in rodent models of migraine. It also needs to be clarified why histamine is so effective in provoking migraine attacks, but antihistamines are not effective in migraine. It remains unclear how the different signaling molecules interact, but it seems obvious that migraine is not just explained by the action of CGRP or one of the other signaling molecules. Here more human and animal experimental studies are clearly needed. Which patients develop migraine with which provoking molecule? Genetic characterization of the patients compared to their response to migraine provoking agents will be interesting. Provocation studies may also help subdividing migraine patients into categories which may or may not respond differently to different antimigraine medications. In other words, provocation studies may prove helpful in developing precision medicine for migraine. Another aspect is the diagnosis of migraine. It is currently based exclusively on clinical features, like psychiatric diseases. But just like the National Institutes of Health in the US have proposed to strengthen psychiatric diagnosis with biomarker studies, migraine provocation may be useful in headache classification. For such studies mass screenings with different provoking molecules are needed. An example to perhaps pursue is the old study of intravenous single injection of a small dose of histamine, which appeared to have a certain diagnostic value [9]. It is probably better than inhalation of histamine which had a high specificity but low sensitivity [49]. GTN has previously been tested as ointment with moderately positive results [5]. It should be examined with IV administration. For that sodium nitroprusside would probably be preferable. Advanced study methods in humans such as PET scanning and different MR modalities have recently been used, but all the provoking substances should have such studies in future. The best animal models of migraine have been developed based on human provocation studies. So far, they have mostly used NO donors. But all provoking agents are in principle valuable tools in animal experimental studies. They are indispensable for the understanding of the mechanisms of action of migraine provoking substances. Finally, the already documented provoking substances in migraine should be applied to cluster headache, tension type headache and perhaps to other types of headaches to better understand the mechanisms of these diseases. In conclusion, the catalog of needed future research is enormous, and only the limited resources currently allocated to headache research limits the possibilities. Provocation studies have pointed to several promising migraine drug targets. Only the restricted investment by the pharma industry limits the possibilities for novel migraine drugs. The positive examples of anti CGRP and anti PACAP treatments should hopefully stimulate the industry to take more action.

## Data Availability

No datasets were generated or analysed during the current study.
